# Crystal chemistry and single-phase synthesis of Gd^3+^ substituted Co–Zn ferrite nanoparticles for enhanced magnetic properties†

**DOI:** 10.1039/c8ra04282a

**Published:** 2018-07-16

**Authors:** R. A. Pawar, Sunil M. Patange, A. R. Shitre, S. K. Gore, S. S. Jadhav, Sagar E. Shirsath

**Affiliations:** Department of Physics, Arts, Commerce and Science College Satral 413711 MS India; Materials Science Research Laboratory, SKM Gunjoti Osmanabad 413613 MS India; Department of Physics, Yashwantrao Chavan Mahavidyalaya Tuljapur Osmanabad 413601 MS India; Dnyanopasak Shikshan Mandal's Arts, Commerce and Science College Jintur 431509 India; School of Materials Science and Engineering, The University of New South Wales NSW 2052 Sydney Australia shirsathsagar@hotmail.com s.shirsath@unsw.edu.au +61 469029171

## Abstract

Rare earth (RE) ions are known to improve the magnetic interactions in spinel ferrites if they are accommodated in the lattice, whereas the formation of a secondary phase leads to the degradation of the magnetic properties of materials. Therefore, it is necessary to solubilize the RE ions in a spinel lattice to get the most benefit. In this context, this work describes the synthesis of Co–Zn ferrite nanoparticles and the Gd^3+^ doping effect on the tuning of their magnetic properties. The modified sol–gel synthesis approach offered a facile way to synthesize ferrite nanoparticles using water as the solvent. X-ray diffraction with Rietveld refinement confirmed that both pure Co–Zn ferrite and Gd^3+^ substituted Co–Zn ferrite maintained single-phase cubic spinel structures. Energy dispersive spectroscopy was used to determine the elemental compositions of the nanoparticles. Field and temperature dependent magnetic characteristics were measured by employing a vibration sample magnetometer in field cooled (FC)/zero field cooled (ZFC) modes. Magnetic interactions were also determined by Mössbauer spectroscopy. The saturation magnetization and coercivity of Co–Zn ferrite were improved with the Gd^3+^ substitution due to the Gd^3+^ (4f^7^)–Fe^3+^ (3d^5^) interactions. The increase in magnetization and coercivity makes these Gd^3+^ substituted materials applicable for use in magnetic recording media and permanent magnets.

## Abstract 2

Rare earth (RE) ions are known to improve the magnetic interactions in spinel ferrites if they are accommodated in the lattice, whereas the formation of a secondary phase leads to the degradation of the magnetic properties of materials.

## Graphical abstract

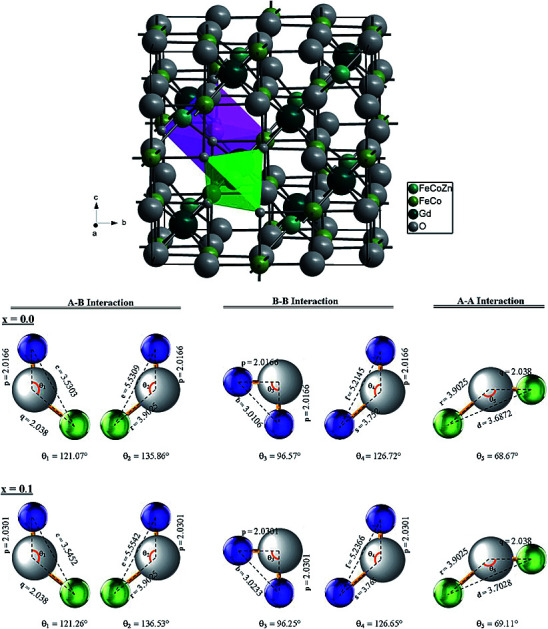

## Introduction

Due to its remarkable electromagnetic properties and physical/chemical stability, cobalt–zinc (Co–Zn) spinel ferrite has attracted lots of interest across the research community.^1^ It has applicability as an active material in catalysis^2,3^ supercapacitive energy storage,^4^ and microwave absorption.^5^ Furthermore, it is applicable in bio-medical fields due to its considerable magneto-crystalline anisotropy, high Curie temperature and moderate saturation magnetization at room temperature. The cation distribution among tetrahedral (A) and octahedral [B] sites in Co–Zn ferrites can be written as (Zn_1−*x*_Fe_*x*_)_A_ [Co_*x*_Fe_2−*x*_]_B_ O_4_. Fe^3+^ ions migrate from (A) to [B] sites with the substitution of Zn^2+^ in Co ferrite, and consequently the Fe_A_–O–Fe_B_ interaction becomes less significant.^6^ The use of zinc substitution can enhance the saturation magnetization up to a certain concentration, but it suppresses the values of the anisotropy constant.^7–9^

Rare earth (RE) elements have large ionic radii, and when they are substituted into the spinel lattice, they may drive the cell symmetry to change by generating internal stress. It is well known that interactions between Fe–Fe ions (spin coupling effect of 3d electrons) govern the magnetic interactions and electrical resistivity of ferri/ferro-magnetic oxides. Therefore, by introducing RE ions into the spinel crystal lattice, an interaction between Fe–RE ions occurs (3d^5^–4f^7^ coupling) which leads to changes in both the magnetic and electrical characteristics of the ferrites.^10–12^ Strong spin (S)–orbit (L) coupling in Co^2+^ ions is responsible for generating large magneto-crystalline anisotropy in CoFe_2_O_4_, and 4f^7^ grouped RE ions (RE^3+^) possess similar spin (S)–orbit (L) coupling. RE^3+^ ions can be stabilized in B-sites of the Co–Zn spinel crystal lattice and could be responsible for the migration of Co^2+^ (3d^7^) ions from the octahedral to the tetrahedral sites with a magnetic moment aligned anti-parallel to those of the RE^3+^ ions in the spinel lattice. This would be expected to significantly modify the magnetic moment. Furthermore, the anisotropy energy constant and ferri/ferro-magnetic ordering temperature of the Co–Zn spinel structured ferrite can be tuned with RE^3+^ substitution. Among the RE^3+^ ions, gadolinium (Gd^3+^) possesses the magnetic moment of 8 *μ*_B_,^13^ and has a single ion anisotropy of approximately zero with a spherically symmetrical charge distribution of 4f^7^.^14–19^

However, there are challenging issues with RE substituted compounds, including the maintenance a single-phase cubic spinel structure and understanding the magnetism of the complex. RE ions have a low solubility limit in spinel ferrite and form a secondary orthoferrite-phase RFeO_3_ beyond their limit.^6,20,21^ RE ions improve the magnetic properties if they are accommodated in the spinel lattice whereas the formation of a secondary phase leads to the degradation of the magnetic properties of the materials. Therefore, it is necessary to solubilize the RE ions in the spinel lattice to get the most benefit. One of the ways to solubilize the RE ions in the spinel lattice is to sinter the material with high temperature. However, sintering at high temperature is not the best option for practical applications. The sol–gel approach has advantages over other methods, owing to the short preparation time, good stoichiometric control over the prepared sample, and inexpensive precursors.^22,23^ A proper tuning of the nitrate to fuel ratio can generate a high temperature during the combustion process. This temperature can easily solubilize the RE ions in the spinel lattice. Furthermore, it creates nanoparticles of a controlled size and a defined morphology. Therefore, in this work an environmentally friendly, facile, modified sol–gel approach, which uses water as the only solvent was used to synthesize single-phase ferrite nanoparticles. We slightly modified the sol–gel method by delaying the combustion to allow sufficient time for the elements to react properly with each other.

In this work, our aim was to synthesize the Gd^3+^ substituted Co–Zn spinel ferrite by a sol–gel auto-combustion process for the formation of a single-phase compound. The complex magnetic nature of this compound was studied to understand its temperature and magnetic field dependent magnetic properties.

## Experimental

**Fig. 1:**
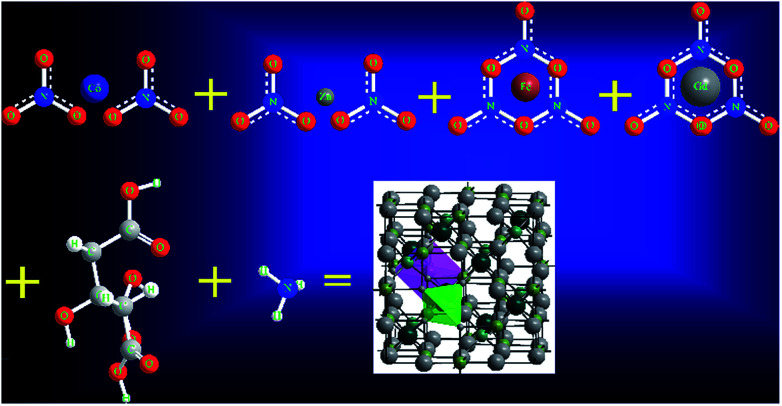
Chemical reaction to synthesize Co_0.7_Zn_0.3_Gd_*x*_Fe_2−*x*_O_4_ by the sol–gel auto-combustion method.

The Gd^3+^ substituted Co–Zn ferrite with the formula Co_0.7_Zn_0.3_Gd_*x*_Fe_2−*x*_O_4_ was prepared by a sol–gel auto combination method. Analytical grade cobalt nitrate (Co(NO_3_)_2_·6H_2_O) (Sigma-Aldrich, 99%), zinc nitrate (Zn(NO_3_)_2_·6H_2_O) (Sigma-Aldrich, 99%), gadolinium nitrate (Gd(NO_3_)_3_·6H_2_O) (Sigma-Aldrich, 99.9%), iron nitrate (Fe(NO_3_)_3_·9H_2_O) (Sigma-Aldrich, 99.95%) and citric acid (C_6_H_8_O_7_·H_2_O) (Sigma-Aldrich, 99%) were used as the starting materials and were dissolved in water. The metal nitrate to citric acid ratio was kept at 1 : 3 and then the pH of the mixed solution was kept at 7 by adding ammonia (NH_3_) solution. The solution was heated to 100 °C and then stirred. After 2 h of continuous heating and stirring, the solution converted into a viscous gel. This formed gel was kept in an air-tight compartment for 24 h at room temperature in order to allow the elements to react with each other. The gel was then heated at 150 °C until it automatically converted into a fluffy powder by self-combustion. Finally, the as-prepared powder was annealed at 650 °C for 5 h. The chemical reaction to prepare the Gd^3+^ substituted Co–Zn ferrite is shown in Fig. 1.

The phase formation in the prepared samples was characterized by X-ray diffraction (XRD, Philips X'Pert instrument) with Cu-Kα radiation (wavelength *λ* = 1.54056 Å) at room temperature. The particle size was investigated by transmission electron microscopy (TEM) (JEOL 3010). Magnetic hysteresis was measured at 10 and 300 K using a vibrating sample magnetometer. Field cooled (FC) and zero field cooled (ZFC) measurements with an external applied magnetic field of 500 Oe were carried out in the temperature range of 10–375 K. ^57^Fe Mössbauer measurements were carried out in transmission mode with a ^57^Co radioactive source in constant acceleration mode using a standard PC-based Mössbauer spectrometer equipped with a Wissel velocity drive. Velocity calibration of the spectrometer was done with a natural iron absorber at room temperature. The spectra were analyzed with the NORMOS program, considering the distribution of hyperfine fields.

## Results and discussion

### Crystal structure and phase identification

**Fig. 2:**
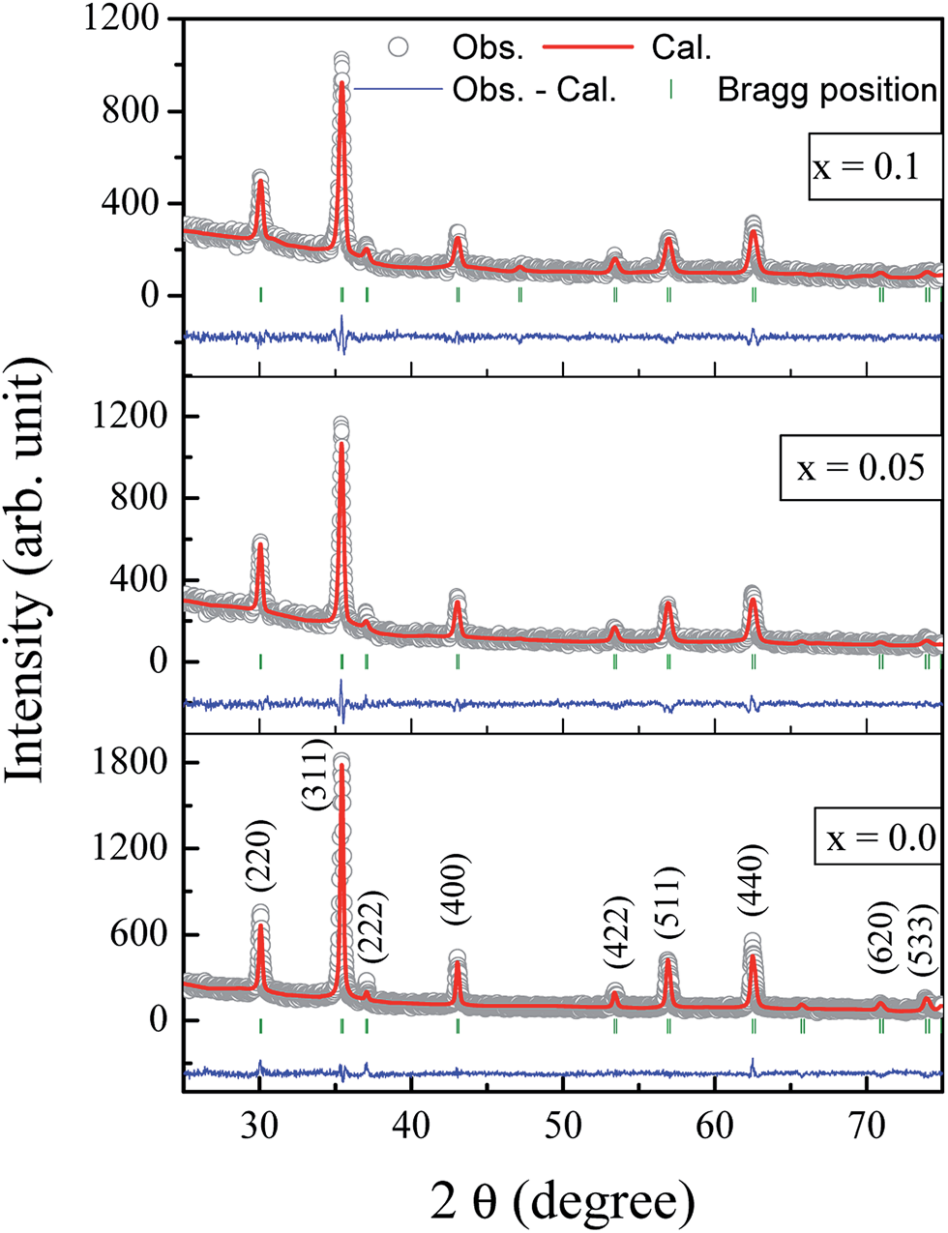
Rietveld refined X-ray diffraction patterns of Co_0.7_Zn_0.3_Gd_*x*_Fe_2−*x*_O_4_ (*x* = 0.0, 0.05, 0.1). The Bragg peak positions are shown in green at the bottom of the XRD patterns. These Bragg peaks are correlated to the space group *Fd*3*m* for Co_0.7_Zn_0.3_Gd_*x*_Fe_2−*x*_O_4_.

**Fig. 3:**
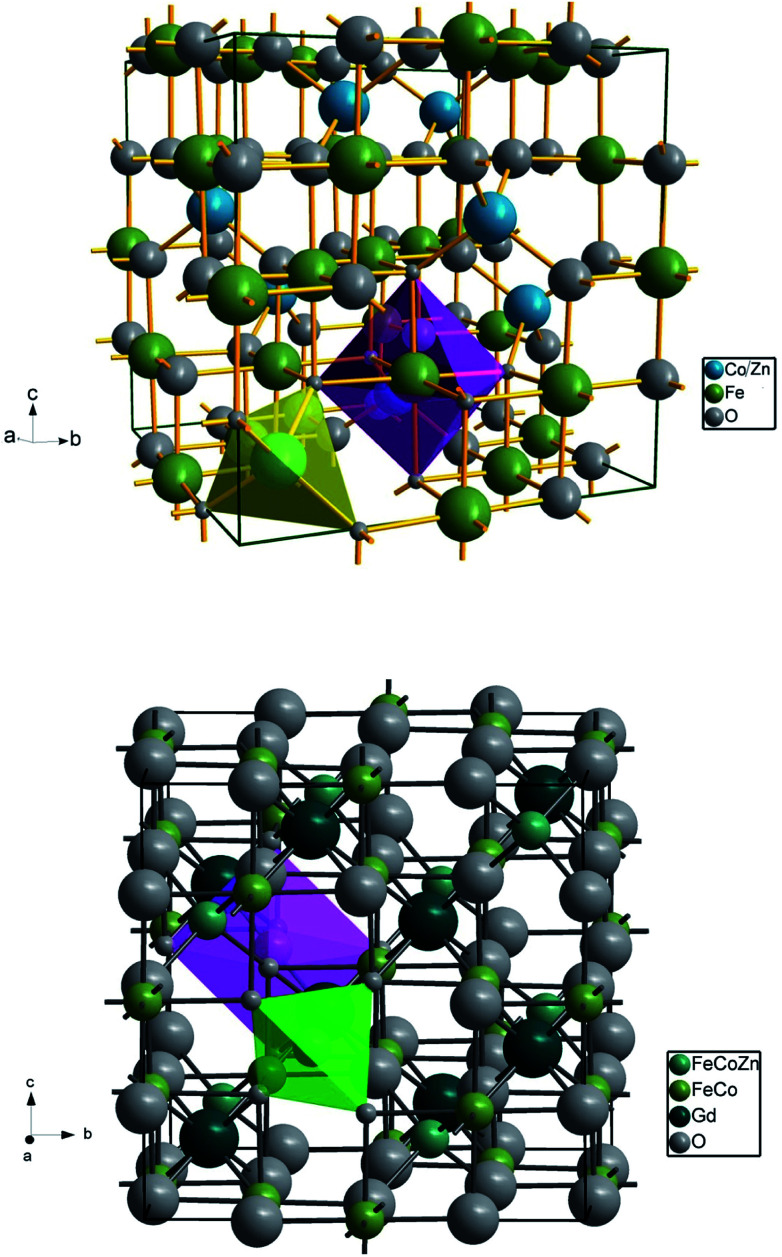
Cubic spinel crystal structures of Co_0.7_Zn_0.3_Fe_2_O_4_ and Co_0.7_Zn_0.3_Gd_0.1_Fe_1.9_O_4_. The spinel structure belongs to the space group *Fd*3*m*. The cubic unit cell is formed by 56 atoms: 32 oxygen anions are dispersed in a cubic close packed structure that nearly forms an fcc lattice, with many interstitial positions partially occupied by tetrahedral-A and octahedral-B atoms; and 24 cations occupy 8 of the 64 A-sites and 16 of the 32 B-sites. The yellow/green and pink shaded areas correspond to the tetrahedral and octahedral sites respectively.

The Rietveld refined X-ray diffraction patterns of typical Co_0.7_Zn_0.3_Gd_*x*_Fe_2−*x*_O_4_ compositions with *x* = 0.0, 0.05 and *x* = 0.1 are shown in Fig. 2. The FullProf program was used for the structural refinements. Obtained Rietveld refined parameters such as the weighted/unweighted profile R-factor (*R*_wp_/*R*_p_), expected *R* factor (*R*_exp_) and goodness fit factor (*χ*^2^) are given in Table 1. The crystal structures of the un-doped and most highly doped Co–Zn ferrites in the presently investigated system (*x* = 0.0 and *x* = 0.1, respectively) were obtained from the Rietveld refinement; they are given in Fig. 3 and are correlated with Gd^3+^ substitution. Details of the refinement are given in the ESI.†

**Table 1:** Discrepancy factors (*R*_wp_), expected values (*R*_exp_), goodness fit factors (*χ*^2^), and crystallite sizes (*t*_xrd_) of Co_0.7_Zn_0.3_Gd_*x*_Fe_2−*x*_O_4_

Comp. *x*	*R*_wp_	*R*_exp_	*χ* ^2^	*t*_xrd_ (nm)
0.0	8.38	8.38	1.00	34
0.025	8.25	8.72	1.12	31
0.05	8.69	9.37	1.16	32
0.075	7.89	8.03	1.04	28
0.1	7.49	7.49	1.00	25

The formation of a single-phase cubic spinel structure without any trace of a secondary phase of GdFeO_3_-orthoferrite is confirmed by the XRD pattern. These results confirmed that the sol–gel method used for the synthesis of these samples successfully pushed the Gd^3+^ ions into the Co–Zn spinel ferrite matrix over the entire range of Gd^3+^ substitution levels. It is a known fact that the secondary-phase formation of orthoferrite is mainly governed by the electronic configuration, the larger ionic radii of the RE^3+^ ions and their diffusion at the grain boundaries. Further, Gd^3+^–O^2−^ has a larger bond energy than Fe^3+^–O^2−^, and therefore Gd^3+^ ions require more energy to enter into the lattice to form Gd^3+^–O^2−^ bonds.^24^ It is noteworthy that the Gd^3+^ substituted Co–Zn ferrite samples may require more energy for complete crystallization and grain growth because of the higher thermal stability of Gd^3+^ ions than that of pure Co–Zn spinel ferrite. Therefore, a high temperature is generally required for the RE ions with their large ionic radii to enter into a cubic spinel matrix for the formation of single phase. It is worth mentioning here that the samples were sintered at the relatively low temperature of 600 °C, though the heat generated during the combustion process was mainly responsible for producing the single phase of the larger Gd^3+^ ion substituted Co–Zn ferrite.

The extrapolation function F(*θ*), *i.e.*, the Nelson–Riley function, for each reflection of the studied sample was calculated to obtain the lattice constant:^25^1
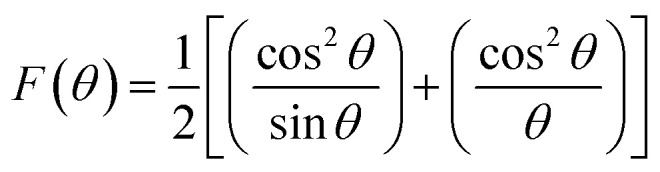


The relation is represented as a straight line for each value of *x*. The true values of the lattice parameter can easily be obtained by extrapolating the line to the value F(*θ*) = 0 or *θ* = 90°.

The lattice constant (*a*) increased from 8.403 to 8.409 Å (±0.002 Å) with the incorporation of Gd^3+^ ions into the Co–Zn ferrite and the behavior was linear throughout the Gd^3+^ substitution range confirming the occupancy of Gd^3+^ ions in the Co–Zn ferrite spinel matrix. The cationic radii of the substituent ions replacing Fe^3+^ ions in the spinel lattice matrix govern the crystal lattice size. In the present case, the ionic radius of the Gd^3+^ ion (0.94 Å) is larger than that of the Fe^3+^ ion (0.67 Å), and therefore it is responsible for increasing the lattice constant.

The root mean square (rms) lattice strain that developed during the sintering treatment and Gd^3+^ substitution was obtained from the full-width-at-half-maximum (FWHM) values of the XRD peaks using the Williamson–Hall (W–H plot) method. The obtained slopes of all the Williamson–Hall plots were negative (figure not shown here) indicating fine grain size samples experiencing compressive strain. The observed results can be related to the occupancy of Fe ions in the tetrahedral and octahedral sites. An increase in occupancy by the larger Gd^3+^ ions in the Co–Zn ferrite matrix and the migration of Fe ions from A to B sites gives rise to compressive strain in the nanoparticles resulting in a smaller distance between the B site ions (2.9731 Å, *x* = 0.1) than that for the A site ions (3.6413 Å, *x* = 0.1).

**Fig. 4:**
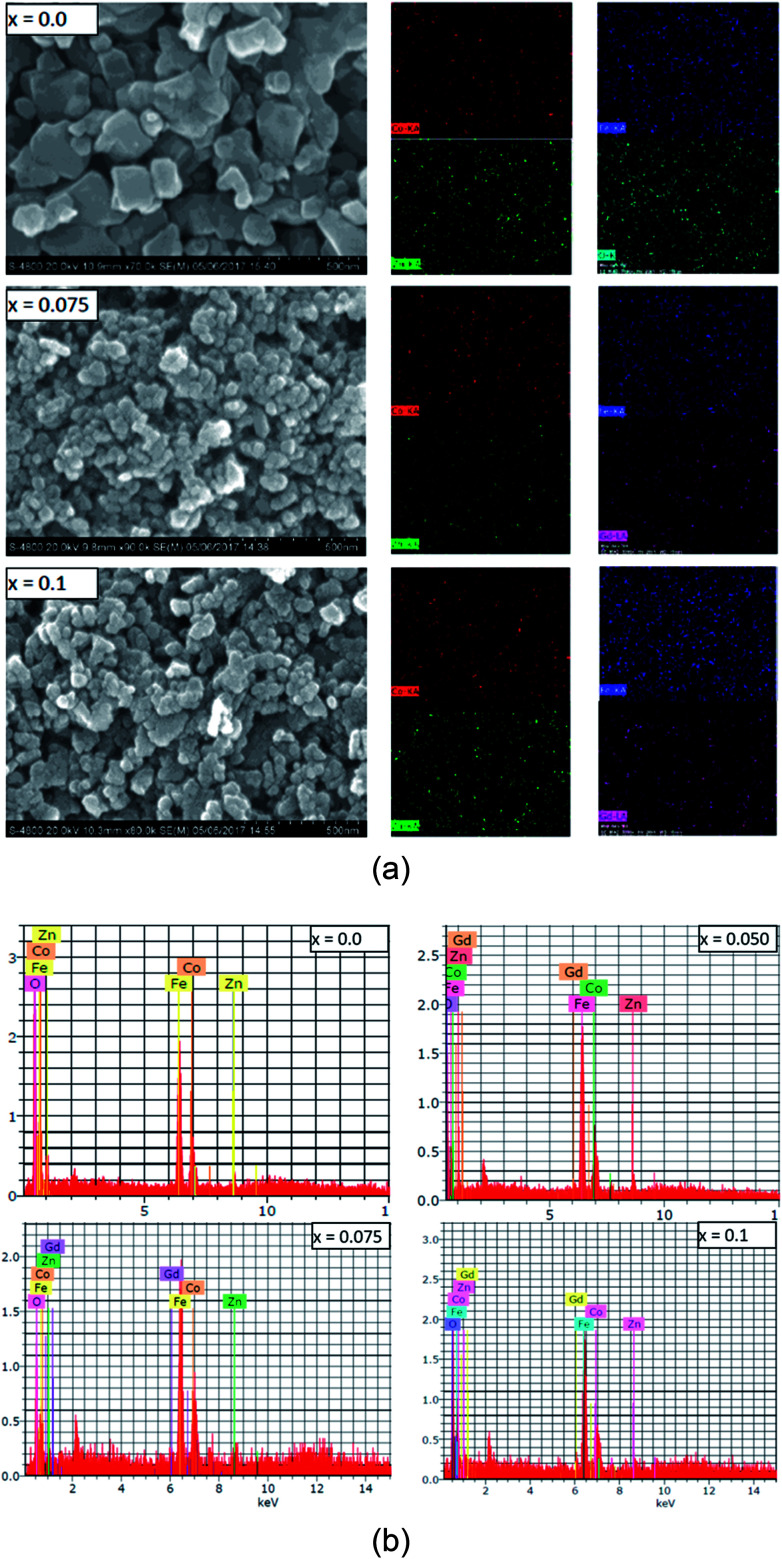
(a) Scanning electron micrographs and the respective color mapping analyses (where Co, Fe, Zn, Gd and O elements are represented by red, blue, green and pink colors, respectively) of Co_0.7_Zn_0.3_Gd_*x*_Fe_2−*x*_O_4_, (b) energy dispersive X-ray spectra of Co_0.7_Zn_0.3_Gd_*x*_Fe_2−*x*_O_4_.

The grain and surface morphology of the sol–gel synthesized samples were examined using scanning electron microscopy (SEM). SEM images of three typical samples with *x* = 0.0, 0.075 and 0.1 are presented in Fig. 4(a). It can be observed from Fig. 4(a) that the shapes of the grains are not regular, however they are evenly sized with evidence of pores around the grain clusters. The morphology of the samples is slightly changed with the Gd^3+^ substitution. It can be observed from the SEM images that most of the grains are bound to each other. This may be due to the annealing treatment that causes agglomeration in the magnetic Gd^3+^ substituted Co–Zn ferrite powder. It could also be an indication that the prepared samples possess better magnetic properties than the Co–Zn ferrite.

SEM mapping is demonstrated in Fig. 4(a). Co, Zn, Fe, Gd and O ions are all distributed uniformly. Energy dispersive X-ray analysis (EDAX) was carried out to obtain the elemental stoichiometry and to support our investigation into the Co_0.7_Zn_0.3_Gd_*x*_Fe_2−*x*_O_4_ ferrite. EDAX of representative samples is given in Fig. 4(b). As expected, the Fe^3+^ has a very high concentration in the un-doped (*x* = 0.0) Gd^3+^ ions and it decreases with the substitution by Gd^3+^. The elemental analysis determined from EDAX is analogous to the starting proportions. The quantification from EDAX is consistent with that expected due to the surface crystalline defects of the nanoparticles which explains the difference between the theoretical and experimental values for the atomic ratio. The crystallite size (*t*_xrd_) of the obtained nanoparticle samples was determined from Scherrer's equation.^26^ As observed from the XRD patterns and Table 1, the *t*_xrd_ values decreased from 34 to 25 nm with increasing substitution by Gd^3+^.

**Fig. 5:**
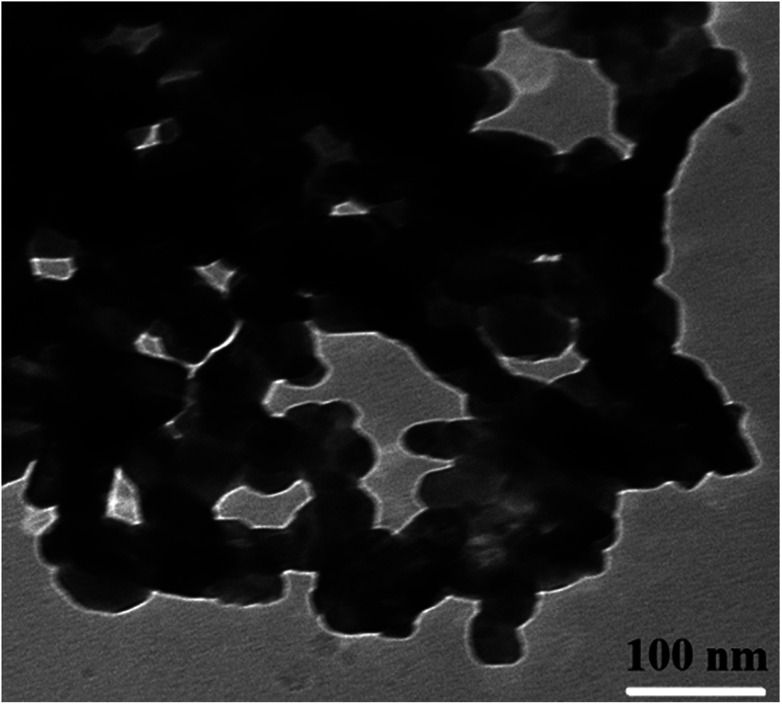
TEM image of a typical sample (*x* = 0.0) of Co_0.7_Zn_0.3_Gd_*x*_Fe_2−*x*_O_4_.

TEM observations were carried out in order to estimate the exact particle size (Fig. 5). The shapes of the nanoparticles observed from the TEM image are regular and uniform but show partial agglomeration. The obtained particle size is decreased from 40 to 27 nm with the substitution of Gd^3+^ ions into Co–Zn ferrite. Rezlescu *et al.* have also observed the reduction in grain/particle size and microstructure variations in ferrites upon substitution by RE ions with their higher ionic radii.^27^ Similar results with grain size reduction were observed in Gd^3+^ substituted CoFe_2_O_4_ (ref. 28) and MnCrFeO_4_.^29^ Such microstructure variations due to the difference in ionic radii and grain-growth inhibition were also observed in other ceramic materials.^30^ Therefore, it is considered that the Gd^3+^ ions with their higher ionic radii are responsible for the observed decrease in particle size.

Cation distribution plays a vital role in governing the structural, electrical and magnetic properties of AB_2_O_4_ spinel ferrites. There are eight formula units, or a total of 8 × 7 = 56 ions, per unit cell of Co_0.7_Zn_0.3_Fe_2_O_4_ (Fig. 3). The fcc crystal structure is a closed packed arrangement of oxygen ions that each have a large ionic radius of 1.3 Å compared to Co^2+^ (0.745 Å), Zn^2+^ (0.83 Å) and Fe^3+^ (0.67 Å) ions. These cations are distributed over the available spaces in the fcc structure. The spaces are divided into two types, termed tetrahedral A-sites and octahedral B-sites. There are 8 A-sites in which the metal cations are tetrahedrally coordinated with oxygen, and 16 B-sites that possess octahedral coordination. In the present work, Rietveld refinement by the FullProf program was used to estimate the cation occupancies of Co^2+^, Zn^2+^, Fe^3+^ and Gd^3+^ ions. The values obtained for atomic occupancy, and the coordinates are shown in Table 2. Tetrahedral A- and octahedral B-sites are preferentially occupied by Zn^2+^ and Gd^3+^ ions. The ionic radius of a Gd^3+^ ion is 0.94 Å which is large for the tetrahedral site and therefore Gd^3+^ ions are forced to occupy octahedral sites; similarly Co^2+^ ions prefer to occupy octahedral sites because of their site preference energy. Fe^3+^ ions partially migrate from octahedral to tetrahedral sites with the increase in Gd^3+^ substitution. Fig. 3 demonstrates the crystal structures obtained from Rietveld refinement of the un-doped and most highly doped Co–Zn ferrites (*x* = 0.0 and 0.1, respectively). These visual crystal structure demonstrations are very helpful in understanding the occupancy of the constituent ions in the presently investigated ferrite system.

**Table 2:** Values of atomic coordinates (*x*, *y*, *z*) and occupancy (*g*) determined from Rietveld refinements of XRD patterns

Ions	*x* = 0.0		*x* = 0.025		*x* = 0.05		*x* = 0.075		*x* = 0.1	
	*x* = *y* = *z*	Occ (*g*)	*x* = *y* = *z*	Occ (*g*)	*x* = *y* = *z*	Occ (*g*)	*x* = *y* = *z*	Occ (*g*)	*x* = *y* = *z*	Occ (*g*)
Zn	0.1250	0.3000 (1)	0.1250	0.3000 (1)	0.1250	0.3000 (1)	0.1250	0.3000 (1)	0.1250	0.3000 (1)
Fe	0.1250	0.6988 (1)	0.1250	0.6986 (2)	0.1250	0.6988 (2)	0.1250	0.6988 (2)	0.1250	0.6995 (2)
Co	0.5000	0.7000 (1)	0.5000	0.7000 (1)	0.5000	0.7000 (1)	0.5000	0.7000 (1)	0.5000	0.7000 (1)
Gd	0.5000	0.0000	0.5000	0.0249 (1)	0.5000	0.0498 (2)	0.5000	0.0749 (1)	0.5000	0.0999 (1)
Fe	0.5000	1.2999 (2)	0.5000	1.2750 (2)	0.5000	1.2500 (2)	0.5000	1.2250 (2)	0.5000	1.9895 (5)

**Fig. 6:**
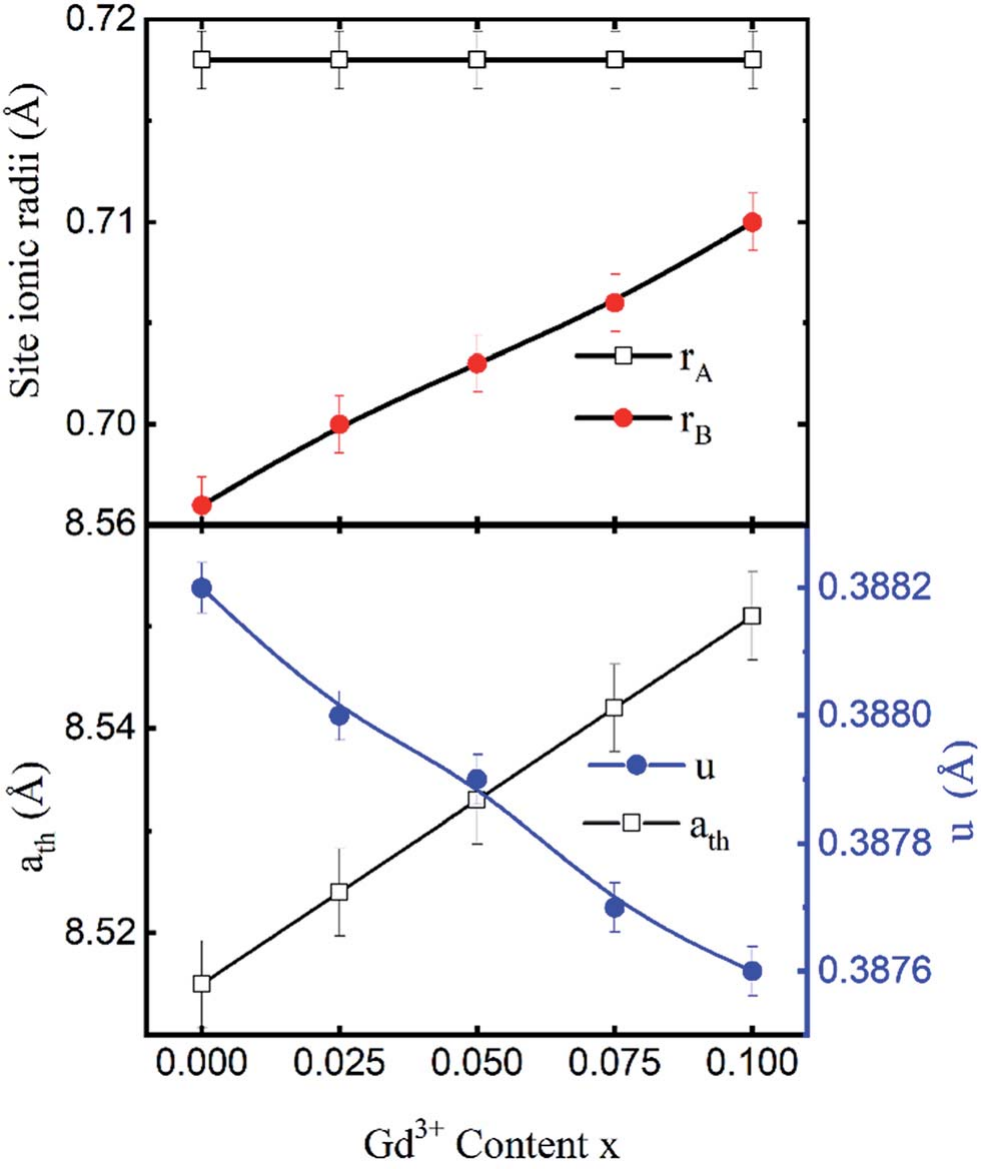
Mean ionic radii at a tetrahedral A-site (*r*_A_) and an octahedral B-site (*r*_B_), theoretical lattice constant (*a*_th_), and oxygen positional parameter (*u*) of Co_0.7_Zn_0.3_Gd_*x*_Fe_2−*x*_O_4_.

The mean ionic radius variations of the tetrahedral A- (*r*_A_) and octahedral B-sites (*r*_B_) are presented in Fig. 6. As observed, *r*_A_ remains almost constant whereas *r*_B_ increases with Gd^3+^ substitution. The increased *r*_B_ is attributed to the occupancy of the larger Gd^3+^ ions at the octahedral B-sites. The theoretical lattice constants (*a*_th_) were determined by using the following equation:^31^2
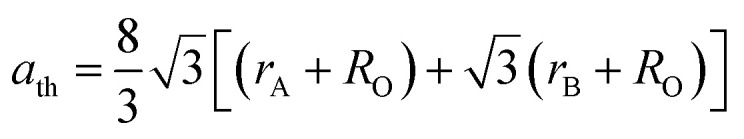
where *R*_O_ is the radius of the oxygen ion. Like the experimentally observed lattice constant ‘*a*’, the theoretical lattice constant ‘*a*_th_’ also increased with the Gd^3+^ substitution (Fig. 6). The oxygen positional parameter *u* was calculated using the following equation that measures the distance between a face of a cube and the oxygen ion:^32^3
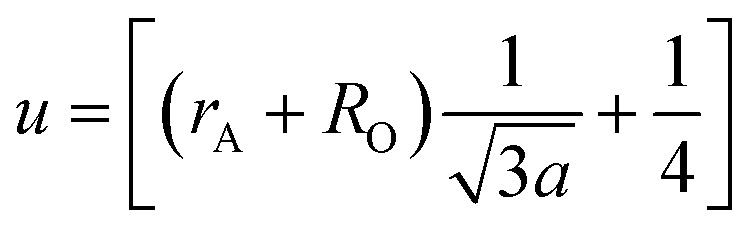


Fig. 6 shows the variation in the oxygen parameter over the entire range of Gd^3+^ substitution levels. In an ideal fcc structure, *u* = 0.375 Å, considering the perfect packing of ions within the crystal lattice. However, the oxygen atoms in the cubic spinel structure are generally not exactly located at the fcc sublattice and therefore cause deformations in positions as evidenced by the oxygen parameter. This also reflects the adjustment of the spinel crystal structure to accommodate ions of different ionic radii at the A and B sublattices. The decreased value of the *u* parameter from 0.3882 Å (*x* = 0.0) to 0.3876 (*x* = 0.1) (which is still a little higher than the ideal value of *u* = 0.375 Å) could be related to the shift of the origin at the tetrahedral sites with the substitution by Gd^3+^ ions at the cost of Fe^3+^ ions at the octahedral sites. Furthermore, the larger Gd^3+^ ions prefer to occupy the octahedral B-sites of the Co–Zn spinel lattice and so remove some of the smaller Fe^3+^ ions from those sites which possibly expand the octahedra-BO_6_ to accommodate the larger Gd^3+^ ion, and subsequently contract the tetrahedra-AO_4_ in the (111) direction.

### Magnetic properties

**Fig. 7:**
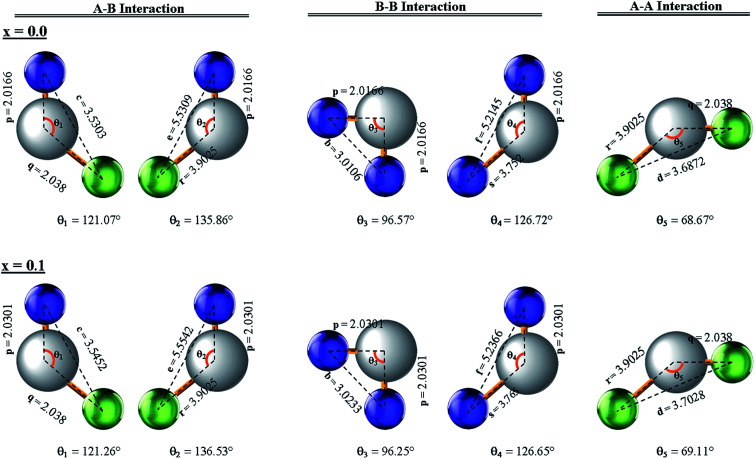
Configurations of ion pairs in spinel ferrites with favourable distances and angles for effective magnetic interactions for *x* = 0.0 and *x* = 0.1.

Three different types of magnetic interactions known as A–A interactions, B–B interactions and A–B interactions are possible between the cations by the super-exchange mechanism through the intermediate oxygen ions (Fig. 7). The interaction energies are negative and hence induce an anti-parallel spin orientation. It is observed that the interaction energies among two magnetic ions (M^I^ and M^II^) depend on the distance of these ions from the oxygen ions through which the interactions occur, and the angle between M^I^–O–M^II^ is represented by *θ*. An angle *θ* of about 180° between the cations gives the maximum interaction energy. The exchange energy decreases rapidly with increasing distance of the cations from the oxygen anion. Out of these three interactions, the A–B interaction is the greatest in magnitude and hence the cation–anion bond lengths are fairly small. The A–A interaction is known to be the weakest interaction as the cation–anion distance is large. The inter-ionic distances between the cations (Me–Me) (*b*, *c*, *d*, *e*, and *f*) and between the cation and anion (Me–O) (*p*, *q*, *r*, and *s*) as well as the bond angles (*θ*_1_, *θ*_2_, *θ*_3_, *θ*_4_, and *θ*_5_) between the cations and cation–anion are calculated using the equations presented in Table 3, and the values are presented in Table 4. As evidenced from Fig. 7 and Table 4, the Me–Me cation distances (*b* to *f*) and distances between the Me–O cation and anion are increased by the Gd^3+^ substitution. The substitution by the larger Gd^3+^ ions for the Fe^3+^ ions causes the BO_6_ octahedra to bulge and results in the increase in the B–O bond length. On the other hand, the tetrahedra-AO_4_ sinks without modifying the 4̄3*m* overall symmetry which could further increase the anion–anion (O–O) distances (bond edges) and therefore result in an increment in the inter-cation distances (Me–Me). It is noted that *θ*_1_ and *θ*_2_ related to the A–B interaction increase, *θ*_3_ and *θ*_4_ related to the B–B interaction decrease, whereas *θ*_5_ corresponding to the A–A interaction slightly increases with the Gd^3+^ substitution. These results suggest that the A–O–B exchange interaction mechanism is strengthened more than those of the A–O–A and B–O–B interactions in the Co–Zn ferrite with the Gd^3+^ substitution, and such an increase in exchange interaction would possibly enhance the saturation magnetization (*M*_s_) values.

**Table 3:** Expressions for determining the cation–cation (Me–Me) and cation–anion (Me–O) distances, and the bond angles

Me–Me	Me–O	Bond angles
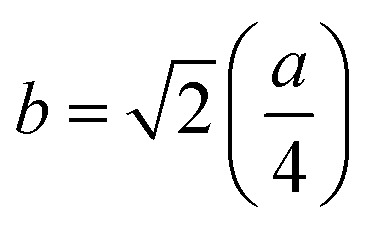	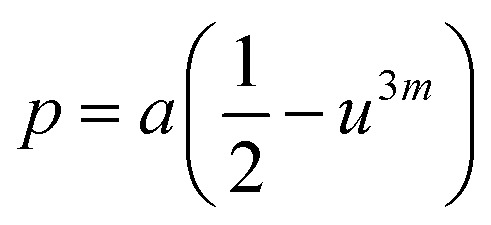	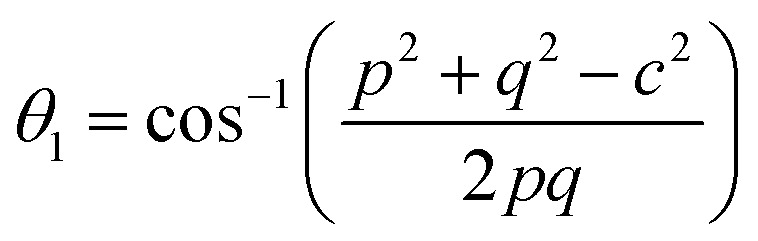
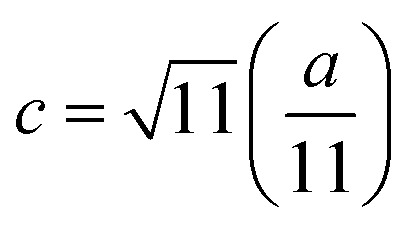	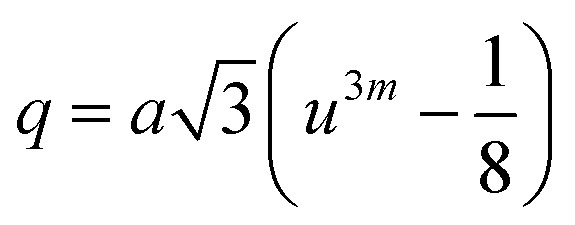	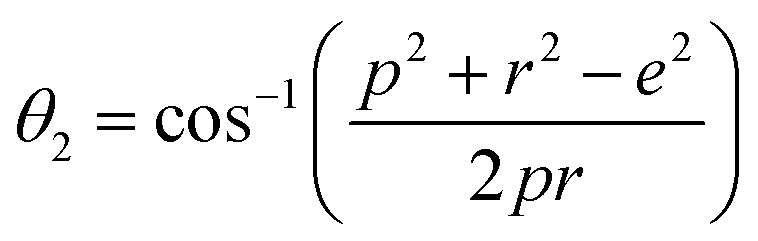
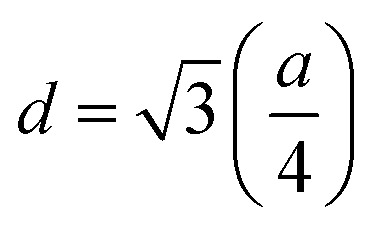	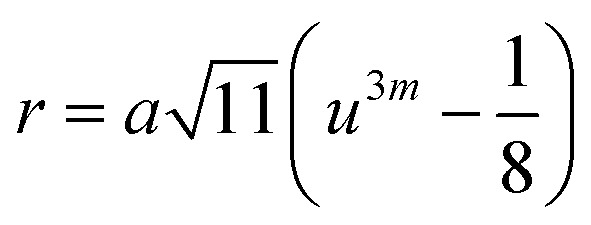	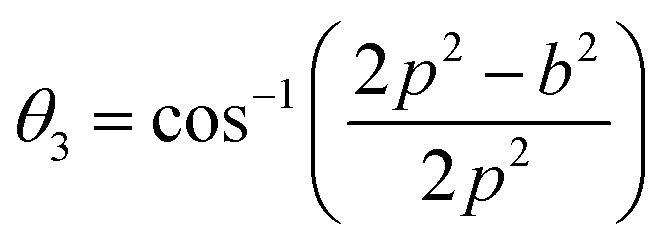
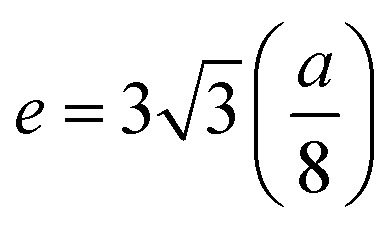	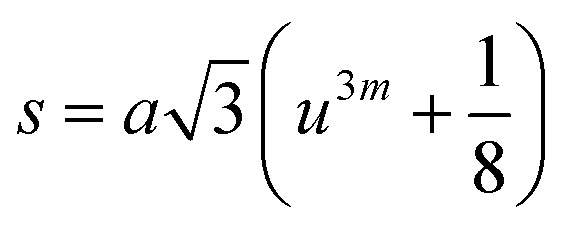	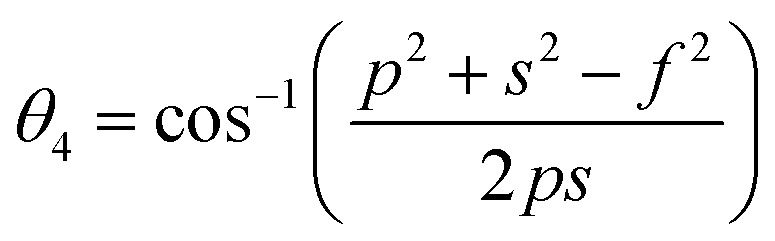
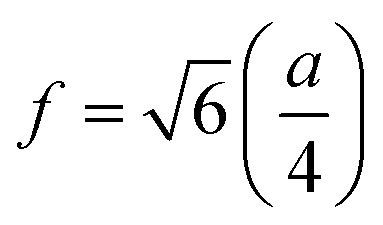		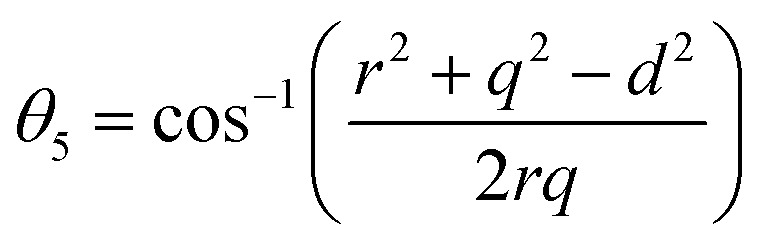

**Table 4:** The calculated inter-ionic distances between cation–anion (Me–O) and cation–cation (Me–Me), and the bond angles for Co_0.7_Zn_0.3_Gd_*x*_Fe_2−*x*_O_4_

Para-meters	*x* = 0.0	*x* = 0.025	*x* = 0.050	*x* = 0.075	*x* = 0.1
*b* (Å)	3.0106	3.0138	3.0170	3.0202	3.0233
*c* (Å)	3.5303	3.5340	3.5377	3.5415	3.5452
*d* (Å)	3.6872	3.6911	3.6950	3.6989	3.7028
*e* (Å)	5.5309	5.5367	5.5426	5.5484	5.5542
*f* (Å)	5.2145	5.2201	5.2256	5.2311	5.2366
*p* (Å)	2.0166	2.0200	2.0234	2.0267	2.0301
*q* (Å)	2.0380	2.0380	2.0380	2.0380	2.0380
*r* (Å)	3.9025	3.9025	3.9025	3.9025	3.9025
*s* (Å)	3.7520	3.7553	3.7585	3.7618	3.7650
*θ*_1_ (°)	121.07	121.12	121.17	121.21	121.26
*θ*_2_ (°)	135.86	136.02	136.19	136.36	136.53
*θ*_3_ (°)	96.57	96.49	96.41	96.33	96.25
*θ*_4_ (°)	126.72	126.70	126.68	126.67	126.65
*θ*_5_ (°)	68.67	68.78	68.89	69.00	69.11

**Fig. 8:**
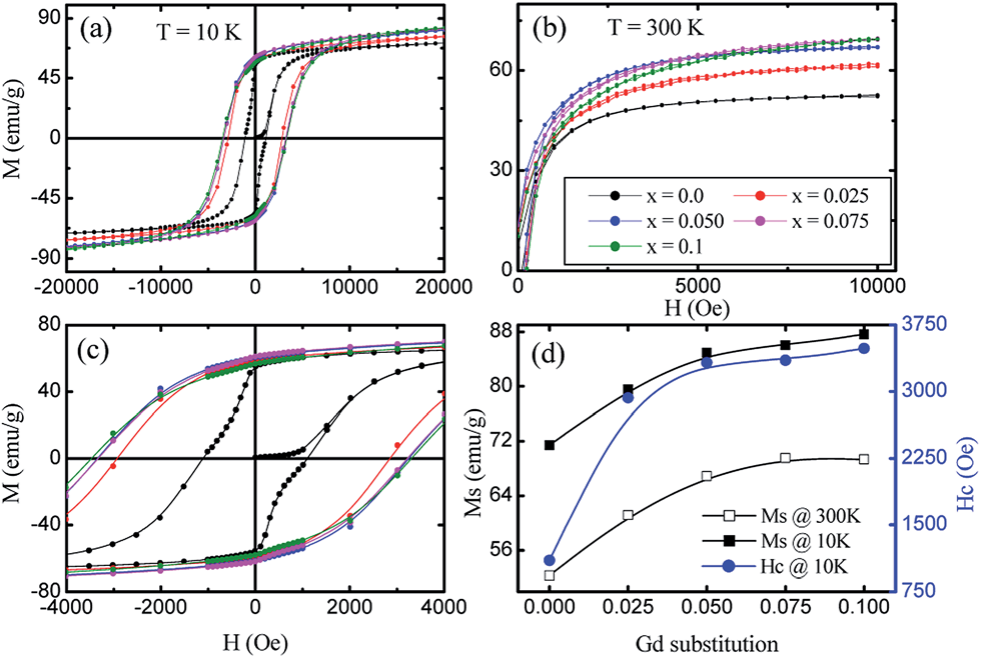
Variation of magnetization (*M*) with applied magnetic field (*H*) of Co_0.7_Zn_0.3_Gd_*x*_Fe_2−*x*_O_4_, measured at (a) 10 K and (b) 300 K, (c) expanded view of lower applied magnetic field of *M*–*H* curve measured at 10 K, (d) variation of saturation magnetization (*M*_s_) with Gd substitution level measured at 10 K and 300 K, and variation of coercivity (*H*_c_) measured at 10 K with Gd substitution level.

The magnetic field dependence characterization was done using a vibrating sample magnetometer at 10 K and room temperature (300 K) by applying magnetic fields of up to 20 and 10 kOe respectively. The so-obtained *M*–*H* curves are presented in Fig. 8(a and b). At room temperature, the very narrow hysteresis loops reveal the soft magnetic material behaviour of the samples, indicating the presence of super-paramagnetic and/or single-domain particles in these ferrites. It can be seen from Fig. 8d that the *M*_s_ values measured at 300 K show an increase from 52.33 emu g^−1^ (*x* = 0.0) to 69.34 emu g^−1^ (*x* = 0.1), whereas, there is an increase from 71.43 emu g^−1^ (*x* = 0.0) to 87.63 emu g^−1^ (*x* = 0.1) for the measurements taken at 10 K. This enhancement in the *M*_s_ with Gd^3+^ substitution could be related to the higher magnetic moment of Gd^3+^ (8 *μ*_B_, 4f^7^ orbital) which resides at octahedral sites as compared to Fe^3+^ (5 *μ*_B_, 3d^5^ orbital). In ferrites, the distribution of cations in tetrahedral A- and octahedral B-sites governs the strength of the exchange interaction among them. Further, these exchange-interactions mainly depend on the bond lengths and bond angles. As discussed earlier, A–B super-exchange interactions between cations at the tetrahedral and octahedral sites are highly dominant when compared to A–A and B–B interactions. There is an enhancement of A-B super-exchange interactions in Co–Zn ferrite with Gd^3+^ substitution, resulting in an enhancement in *M*_s_.

The hysteresis loop measured at 10 K shows the higher value of *M*_s_ as compared to that at 300 K. It is known that thermal energy decreases at lower temperatures causing alignment of magnetic moments parallel to the external magnetic field direction and resulting in an enhancement of saturation magnetization. The mechanism is exactly the opposite at high temperatures where the surface spins experience a few disorder states with similar energies in a short time that weaken their response to the applied magnetic field and thus lower the magnetization. Further, for a ferri/ferro-magnetic bulk system, the saturation magnetization follows the Bloch's law below the Curie temperature:^33^4
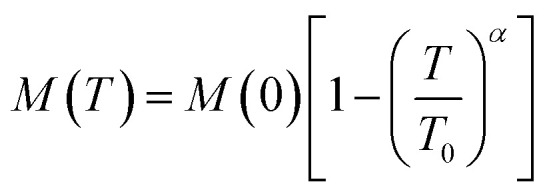
where 
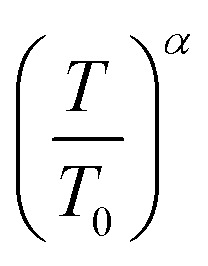
 is Bloch's constant (*B*) that depends upon the crystal structure, *M*(0) is *M*_s_ at 0 K, *T*_0_ is the temperature at which *M*_s_ is zero, *M*(*T*) is the temperature-dependent magnetization, and *α* is the Bloch's exponent (= 3/2). In the low temperature range, Bloch's law is valid for a bulk ferri/ferro-magnetic system due to the availability of a spin-wave excitation mechanism. However, due to the finite size effect, there is a deviation from Bloch's law in the case of thermally dependent magnetization, where the magnons with larger wavelength compared to the particle dimensions do not become excited, and therefore a threshold of thermal energy is necessary for the generation of spin waves in these fine particles. Therefore, a modification in the spin wave structure for the nanoparticle system is carried out in the power law (*T*^*α*^) form where the Bloch's exponent is larger than the bulk counterpart value of 3/2.^34^ The modification of Bloch's law accounts for the confinement effects on the spin-wave spectrum of ferro/ferri-magnetic clusters of various structures that arise due to the presence of a discrete spectrum of spin-wave modes having cut-off wavelengths larger than the size of the system, resulting in an effective exponent (*α*) in Bloch's law which is higher than 3/2 at intermediate temperatures, and a decrement in the saturation magnetization at very low temperatures.^35^ This modification in Bloch's law is valid for the temperature range of 50–300 K.^36,37^

Therefore, in the present study, the increase in *M*_s_ of the Gd^3+^ substituted Co–Zn ferrite nanoparticle system at 10 K can be attributed to: (i) the contribution from the shell-spin moment to the resultant magnetization at low temperatures,^38^ (ii) the quantization of the spin-wave spectrum resulting from the finite-size effect due to the energy gap in the spin-wave spectrum of the nanoparticles, and (iii) the possible contributions from the paramagnetic-Zn^2+^ ions that can be activated at low temperatures.

Fig. 8c shows a magnified view of the low magnetic field region to make the coercivity (*H*_c_) more visible at different levels of Gd^3+^ substitution. A large increase in coercivity at 10 K as compared to 300 K is observed. The increase in *H*_c_ at 10 K can be explained on the basis of the thermal energy in the blocked/frozen moment being insufficient to overcome the magnetic anisotropy barrier. In the case of a non-interacting 3D single domain magnetic nanoparticle assembled with uniaxial magnetic anisotropy, the *H*_c_ in the temperature range (0–*T*_B_) can be modified by the thermal activation of a particle's moment across the anisotropy barriers according to the following equation:^39,40^5
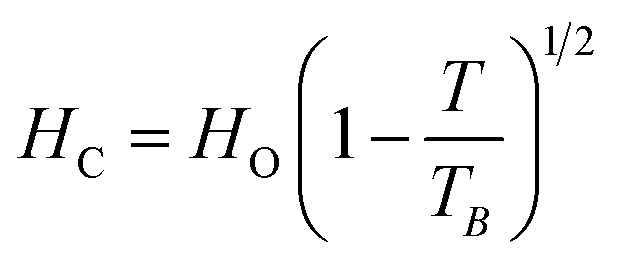
where *H*_O_ is the coercivity at *T* = 0 K, and can be determined by extrapolating the curve of *H*_c_*vs.* temperature towards the field axis, while *T*_B_ is the super-paramagnetic blocking temperature of the nanoparticles. It is evident from eqn (5) that at low temperatures, the enhancement of anisotropy is mainly responsible for the increase in *H*_c_. Apart from this, the intrinsic structural properties, including the randomness of the anisotropy axes, the volume distribution and the interparticle interactions may contribute to the thermal dependence variation of *H*_c_.^41^ The *H*_c_ of Co–Zn is increased monotonically with Gd substitution which could be attributed to the increased magnetic anisotropy. Furthermore, the increased value of *H*_c_ could be related to the decrease in particle size with Gd^3+^ substitution where demagnetization caused by domain rotation (single domain) requires higher energy compared to the movement of the domain walls (multidomain).^42^

**Fig. 9:**
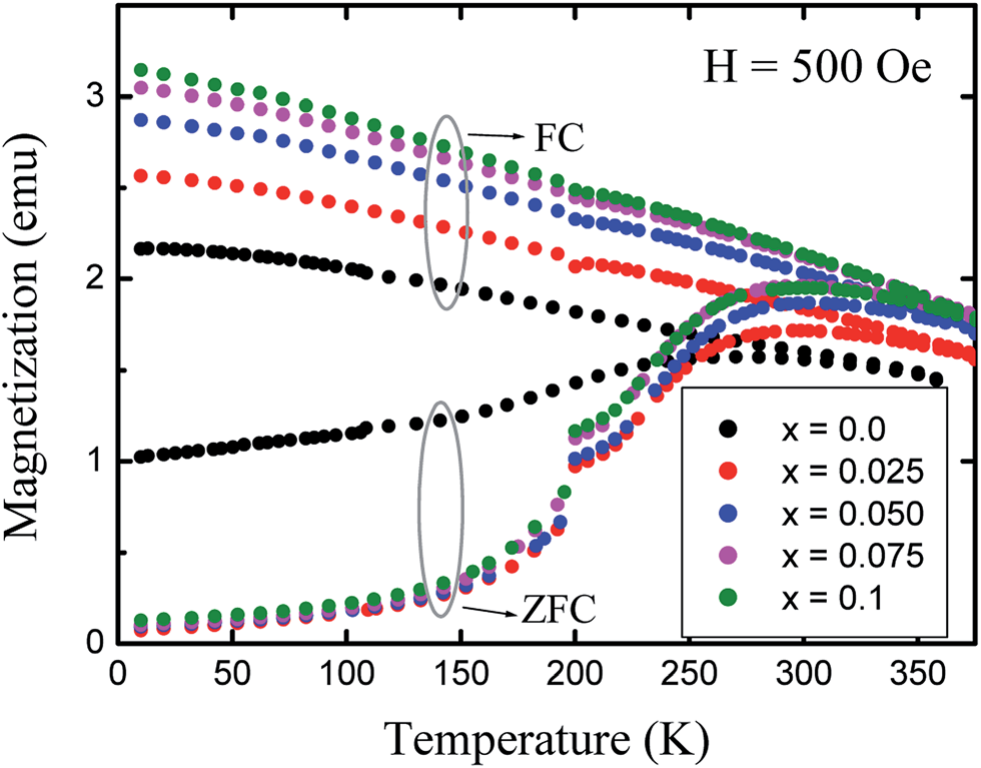
Variation of magnetization (*M*) with temperature (*T*) measured in field cooled (FC) and zero field cooled (ZFC) modes at 500 Oe.

The temperature dependences of the magnetization, in field cooled (FC) and zero field cooled (ZFC) modes, were measured over a temperature range of 10 K to 375 K with the application of a 500 Oe field (Fig. 9). The ZFC curves for all the nanoparticles show peaking behaviour near the blocking temperature (*T*_B_), while the FC increases below *T*_B_. Magnetic nanoparticles form 3D superlattice nanoparticle assemblies because of the strong interactions among the particles, and show a very steady increment. The temperature dependent magnetization behavior is in good agreement with the above discussed Bloch's spin wave model. The large differences in the ZFC and FC curves below *T*_B_ are an indication of the higher coercivity at lower temperature and the differences between the ZFC and FC increase with the level of Gd^3+^ substitution which can be related to the anisotropy constant. It is observed that the blocking temperature of Co–Zn spinel ferrite is modified from 280 K (*x* = 0.0) to 298 K (*x* = 0.1) with the Gd^3+^ substitution. The higher anisotropy associated with the RE Gd^3+^ ions relative to the Fe^3+^ ions causes an overall enhancement of the anisotropy energy that could decrease the probability of ions jumping over the anisotropy barrier and hence increase the blocking temperature.

**Fig. 10:**
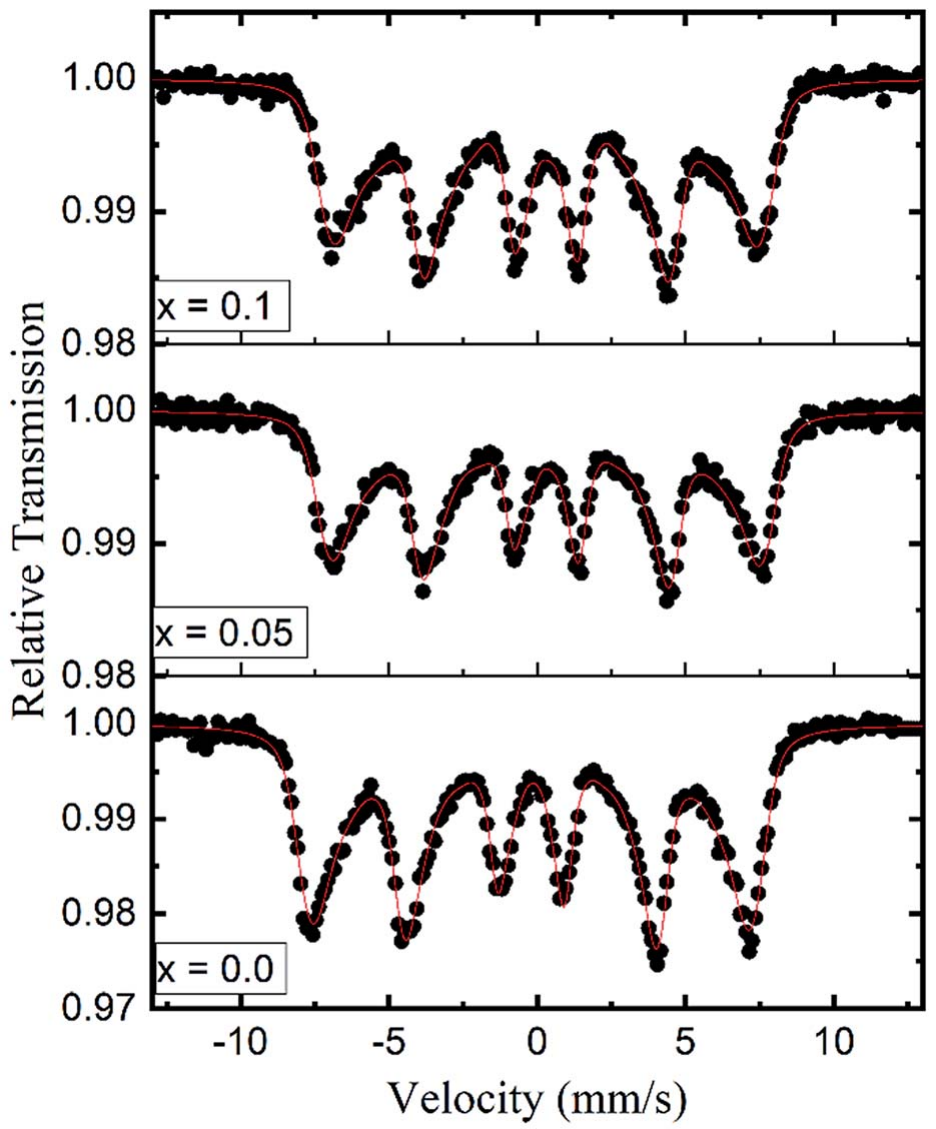
Room temperature Mössbauer spectra for the typical samples (*x* = 0.0, 0.05 and 0.1) of Co_0.7_Zn_0.3_Gd_*x*_Fe_2−*x*_O_4_.

Room temperature Mössbauer spectra of typical samples *x* = 0.0, 0.05 and 0.1 are shown in Fig. 10. It is obvious that the Mössbauer spectra of all the samples exhibit well-defined Zeeman split sextets, due to Fe^3+^ ions at the tetrahedral sites and Fe^3+^ ions at the octahedral sites. The broadened six-line pattern may arise from the randomly distributed magnetic (Fe^3+^, Co^2+^ and Gd^3+^) and paramagnetic (Zn) ions in the available A and B sublattices. Interestingly, there is no central paramagnetic contribution from the paramagnetic-Zn ions in the Mössbauer spectra. This means that the local paramagnetic-Zn^2+^ centers are transformed in the ordered magnetic structure and the long-range magnetic interactions surmount the localized paramagnetic interactions by removing the magnetic isolation of the Zn^2+^ ions. The hyperfine field increases from 45.02 to 46.03 tesla with the Gd^3+^ substitution and can be qualitatively explained by Neel's super-exchange interactions.^43^ As both tetrahedral and octahedral sites are occupied by Fe^3+^ ions, so the interaction of Fe^3+^ with Co^2+^ and Gd^3+^ ions is possible. Here, Fe^3+^ ions are replaced by the Gd^3+^ ions which have larger magnetic moments resulting in the strengthening of the magnetic linkages between Fe_A_^3+^–O–Fe_B_^3+^, Fe_A_^3+^–O–Gd_B_^3+^ and Fe_A_^3+^–O–Co_B_^3+^. Consequently Fe^3+^ ions experience an enhancement in the magnetic field at tetrahedral and octahedral sites. It is a known fact that saturation magnetization is directly proportional to the hyperfine field as shown in the present work.

## Conclusions

Gd^3+^ substituted Co–Zn ferrite nanoparticles with a single-phase cubic spinel structure were successfully prepared by a modified sol–gel method. Rietveld refinement of the XRD patterns shows an increase in the lattice constant with Gd^3+^ substitution, confirming the accommodation of the larger Gd^3+^ ions in the Co–Zn ferrite spinel matrix. The estimated cation distribution shows that Co^2+^ and Gd^3+^ ions have strong preferences towards octahedral B-sites, whereas Zn^2+^ ions prefer to occupy tetrahedral A-sites, and Fe^3+^ ions are randomly distributed over the available sites. The theoretical and experimental lattice constants match well with each other. Saturation magnetization studied at 10 K and at 300 K shows an improvement with the Gd^3+^ substitution in the Co–Zn ferrite. Coercivity observed at 10 K shows incremental improvement with Gd^3+^ substitution. The ZFC curves for all the nanoparticles show a peaking behaviour near the blocking temperature, while the FC increases below this temperature. The increment in the anisotropy constant of the Gd^3+^ substituted Co–Zn ferrite results in the increasing bifurcation between the ZFC and FC. The blocking temperature increases from 280 K (*x* = 0.0) to 298 K (*x* = 0.1) with the increase in Gd^3+^ substitution in the Co–Zn ferrite. The Mössbauer spectra of all the samples show typical Zeeman split sextets, related to Fe^3+^ ions at the tetrahedral and octahedral sites. The increases in magnetization and coercivity make these materials applicable for magnetic recording media.

## Conflicts of interest

There are no conflicts to declare.

## Supplementary Material

RA-008-C8RA04282A-s001
