# Supplementary material for: Crystal chemistry and single-phase synthesis of Gd3+ substituted Co–Zn ferrite nanoparticles for enhanced magnetic properties
Source: RSC Adv. 2018 Jul 16;8(44):25258–67. doi: 10.1039/c8ra04282a (PMC9082527; doi:10.1039/c8ra04282a)
Supplement: RA-008-C8RA04282A-s001 [file RA-008-C8RA04282A-s001.pdf]

Electronic Supplementary Material (ESI) for RSC Advances.
This journal is © The Royal Society of Chemistry 2018

# Supplementary information

# Crystal chemistry and single-phase synthesis with higher solubility of $Gd^{3+}$ in Co-Zn ferrite nanoparticles for their enhanced magnetic properties

R. A. Pawar,[a] Sunil M. Patange,[b] A. R. Shitre,[c] S. K. Gore,[d] S. S. Jadhav,[d] and Sagar E. Shirsath*,[e]

[a.] Department of Physics, Arts, Commerce and Science College, Satral 413711, MS, India
[b.] Materials Science Research Laboratory, SKM, Gunjoti, Osmanabad 413613, MS, India
[c.] Department of Physics, Yashwantrao Chavan Mahavidyalaya, Tuljapur, Osmanabad 413601, MS, India
[d.] Dnyanopasak Shikshan Mandal's Arts, Commerce and Science College, Jintur 431509, India.
[d]School of Materials science and Engineering, The University of New South Wales, NSW 2052, Sydney, Australia
* Corresponding Author: Phone: +61 469029171, E-mail: shirsathsagar@hotmail.com: s.shirsath@unsw.edu.au

## Rietveld refinement:

Program refinement of the first samples was started with the space group Fd3m, origin at −3 m, O in 32e-, A-site in 8f and B-site in 16c. In the first step the global parameters, such as 2h-zero and background, were refined. In the next step, the structural parameters such as lattice parameter, atomic coordinates, and site occupancy, were refined. The fitting quality of the experimental data were checked by using the following parameters: the goodness of fit, $\chi^2$; that must tend to one and two reliability factors, $R_{exp}$ and $R_{wp}$ (weighted differences between measured and calculated values) that must be close to or less than 10%.

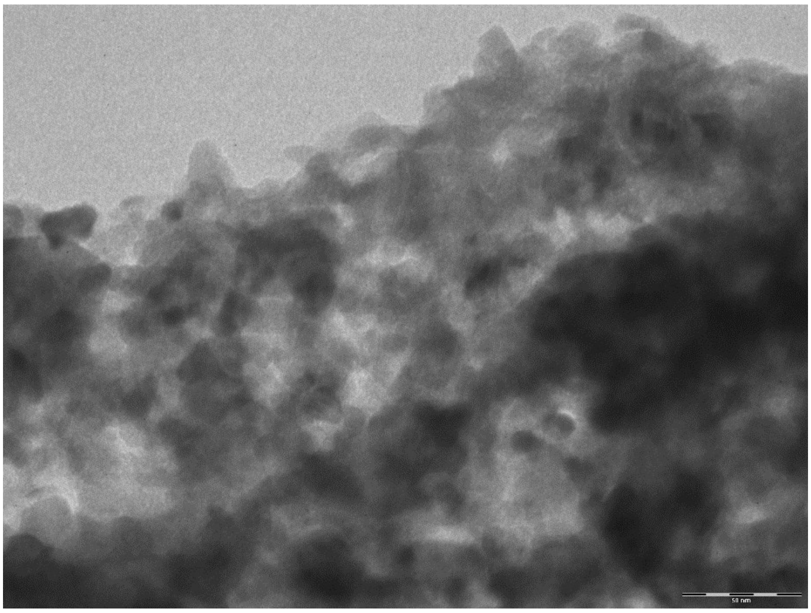

**Supplementary Fig. 1:** TEM images of typical samples (x = 0.1) of $Co_{0.7}Zn_{0.3}Gd_xFe_{2-x}O_4$.
